# Drug Resistance in Cortical and Hippocampal Slices from Resected Tissue of Epilepsy Patients: No Significant Impact of P-Glycoprotein and Multidrug Resistance-Associated Proteins

**DOI:** 10.3389/fneur.2015.00030

**Published:** 2015-02-18

**Authors:** Nora Sandow, Simon Kim, Claudia Raue, Dennis Päsler, Zin-Juan Klaft, Leandro Leite Antonio, Jan Oliver Hollnagel, Richard Kovacs, Oliver Kann, Peter Horn, Peter Vajkoczy, Martin Holtkamp, Heinz-Joachim Meencke, Esper A. Cavalheiro, Fritz Pragst, Siegrun Gabriel, Thomas-Nicolas Lehmann, Uwe Heinemann

**Affiliations:** ^1^Institute of Neurophysiology, Charité Universitätsmedizin Berlin, Berlin, Germany; ^2^Department of Neurosurgery, Charité Universitätsmedizin Berlin, Berlin, Germany; ^3^Laboratorio de Neurologia Experimental, Universidade Federal de São Paulo-Escola Paulista de Medicina, São Paulo, Brazil; ^4^Institute of Physiology and Pathophysiology, University of Heidelberg, Heidelberg, Germany; ^5^Epilepsy Center of Berlin-Brandenburg, Ev. Krankenhaus Königin Elisabeth Herzberge, Berlin, Germany; ^6^Institute of Forensic Medicine – Forensic Toxicology, Charité Universitätsmedizin Berlin, Berlin, Germany

**Keywords:** TLE, surgically resected tissue, seizure-like events, carbamazepine, sodium valproate, phenytoin, verapamil, probenecid

## Abstract

Drug resistant patients undergoing epilepsy surgery have a good chance to become sensitive to anticonvulsant medication, suggesting that the resected brain tissue is responsible for drug resistance. Here, we address the question whether P-glycoprotein (Pgp) and multidrug resistance-associated proteins (MRPs) expressed in the resected tissue contribute to drug resistance *in vitro*. Effects of anti-epileptic drugs [carbamazepine (CBZ), sodium valproate, phenytoin] and two unspecific inhibitors of Pgp and MRPs [verapamil (VPM) and probenecid (PBN)] on seizure-like events (SLEs) induced in slices from 35 hippocampal and 35 temporal cortex specimens of altogether 51 patients (161 slices) were studied. Although in slice preparations the blood brain barrier is not functional, we found that SLEs predominantly persisted in the presence of anticonvulsant drugs (90%) and also in the presence of VPM and PBN (86%). Following subsequent co-administration of anti-epileptic drugs and drug transport inhibitors, SLEs continued in 63% of 143 slices. Drug sensitivity in slices was recognized either as transition to recurrent epileptiform transients (30%) or as suppression (7%), particularly by perfusion with CBZ in PBN containing solutions (43, 9%). Summarizing responses to co-administration from more than one slice per patient revealed that suppression of seizure-like activity in all slices was only observed in 7% of patients. Patients whose tissue was completely or partially sensitive (65%) presented with higher seizure frequencies than those with resistant tissue (35%). However, corresponding subgroups of patients do not differ with respect to expression rates of drug transporters. Our results imply that parenchymal MRPs and Pgp are not responsible for drug resistance in resected tissue.

## Introduction

Patients suffering from partial epilepsies often present with therapeutic difficulties. Up to 80% of patients with temporal lobe epilepsy (TLE) are resistant to treatment with anti-epileptic drugs (AEDs) (1). Drug resistance may depend on the severity of disease (2), development of tolerance (3), changes in drug targets (4–6), network alterations (7, 8), and an open blood brain barrier (9). As drug resistance concerns drugs with different mechanisms of action, the “transporter hypothesis” received considerable attention (10–18). This hypothesis states that drug efflux pumps transport drug molecules out of the brain parenchyma and thereby impede the build-up of tissue AED-concentrations sufficient for seizure control. Multi drug transporter proteins (MDTs) include P-glycoprotein (Pgp) and the multidrug resistance-associated proteins (MRP1-5). Studies in human resected tissue of epilepsy patients show that such proteins are up-regulated or ectopically expressed at the blood brain barrier (19–22) and within the brain parenchyma (13, 18, 23–25).

The aim of our present study is to find out whether Pgp and multidrug resistance-associated proteins (MRPs) in acute hippocampal and temporal cortex slices contribute to drug resistance in the functionally blood brain barrier deprived tissue of drug resistant patients. Our previous study of the dentate gyrus (DG) (26) revealed that induced epileptiform activity in the DG from drug sensitive patients and patients with extra-hippocampal tumors was sensitive to CBZ, while similar activity of drug resistant patients was resistant. Here, we compare effects of drugs, which were “definitely” (phenytoin, PHT), “possibly” (metabolites of carbamazepine, CBZ), and “not” transported (valproate, VPA) by human Pgp (27). Additionally, verapamil (VPM) and probenecid (PBN) were chosen as unspecific inhibitors of Pgp and MRPs because clinical reports described that co-application of VPM with AEDs might restore pharmacosensitivity (28). Moreover, we already presented evidence that the efflux pump function of Pgp and MRPs expressed in slices from resected tissue was altered by VPM and PBN (29). Now, we extend our electrophysiological investigations to the subiculum (SUB) and the temporal neocortex (TCx), and focus on drug effects on induced seizure-like events (SLEs) resembling ictal discharges in intracranial recordings (30).

## Materials and Methods

Surgery specimens were obtained from 72 patients suffering from TLE (years 2005–2009; 58 hippocampal and 52 cortical specimens) of which 65 patients were proven drug resistant, 4 patients were resistant but had adverse effects in at least 1 treatment strategy, and 3 patients were still not “proven” (1 year duration of epilepsy, high seizure frequency or tumor). Pre-surgical analysis including determination of drug resistance was performed in the Epilepsy Center of Berlin Brandenburg (Martin Holtkamp and Heinz-Joachim Meencke) as previously described (26, 31, 32).

All patients underwent a combined resection of the temporal pole with amygdalo-hippocampectomy (TPR+), using a cortical approach. Surgery was a two step procedure. Between 2–2.5 cm (dominant) and 3–3.5 cm (non-dominant) of the temporal pole were removed first, afterwards the mesial temporal structures were resected. The temporal pia mater covering the Sylvian vessels remained intact. After ablation of the temporal pole, the inferior horn was already open or could be readily opened (33). Operations were performed by Thomas-Nicolas Lehmann, Peter Vajkoczy, and Peter Horn in the Department of Neurosurgery, Charité.

For the purpose of the present study, 35 hippocampal and 35 temporal cortex specimens of altogether 51 patients (161 slices in which SLEs were induced) could be included.

The study was approved by the Ethics Committee at the Charité-Universitätsmedizin Berlin (EA125/2001, EA1/042/04) and performed in accordance with the declaration of Helsinki. A written informed consent was obtained from every patient before surgery. Neighboring sections of the tissue in this study were analyzed by the Department of Neuropathology, Charité for diagnostic purposes.

For some patients also the tissue concentrations of OXC, CBZ, and LTG were determined in the Department of Forensic Toxicology, Institute of Forensic Medicine, Charité. CBZ, CBZ-10,11-epoxide, oxcarbazepine, 10-hydroxy-oxcarbazepine, and lamotrigine were determined by high performance liquid chromatography with diode array detector (HPLC-DAD) described by Pragst and colleagues (34). Reference standards for calibration of the five compounds were obtained from LGC Promochem (Wesel, Germany). Between 200 and 500 mg brain tissue were exactly weighed and extracted with the threefold volume of acetonitrile by 2 h incubation and treatment in ultrasonic bath at room temperature. After centrifugation, the acetonitrile layer was completely separated. The residue was thoroughly washed with the same volume acetonitrile and centrifuged again. The extract and the washing were united and the acetonitrile evaporated in a nitrogen stream at 40°C to dryness. The residue was dissolved in 100 μl of the mobile phase (phosphate buffer pH 2.3/acetonitrile 63:37, v/v) and 50 μl were injected for HPLC.

### Tissue transport, preparation, and maintenance

One or two coronal sections of 5 mm thickness were cut from the resected temporal cortex and hippocampus, immediately transferred to cold (1–4°C) carbogenated transport solution containing (in mM) KCl 3, NaH_2_PO_4_ 1.25, glucose 10, MgSO_4_ 2, MgCl_2_ 2, CaCl_2_ 1.6, NaHCO_3_ 21, sucrose 200, and (±) α-tocopherol 0.1 pre-dissolved in ethanol (pH 7.4, osmolality 303 mosmol/kg, 0.005 v% ethanol), and transported to the lab, as previously described (26, 31, 32). Subsequently, the tissue was coronally dissected into slices of 500 μm thicknesses using a vibratome (Campden Instruments Ltd., Leicester, UK). Slices subjected to electrophysiological recordings were immediately transferred to interface chambers, perfused at a rate of 1.7 ml/min with pre-warmed (34.5°C) carbogenated artificial cerebrospinal fluid (ACSF) containing (in mM): NaCl 129, KCl 3, NaH_2_PO_4_ 1.25, glucose 10, MgCl_2_ 2, CaCl_2_ 1.6, NaHCO_3_ 21, (±) α-tocopherol 0.03 (pH 7.4; osmolality 303 mosmol/kg, 0.002 v% ethanol). Recordings commenced 4 h after preparation of slices to permit optimal recovery after surgery and transport in solution with low sodium concentration.

### Electrophysiological recordings and stimulation

Extracellular recordings were performed in the granule cell layer of the DG, in the pyramidal cell layer of the SUB, and in deep layers of the TCx (V–VI), 120–150 μm below the surface of the slice, using double-barreled K^+^-selective/reference microelectrodes as previously described (31). Signals were stored on a computer using Spike2 (version 4.01, CED; Cambridge, UK) with sampling rates of 10 kHz for field potentials (FPs, low pass filter cut-off 3 kHz) and 100 Hz for [K^+^]_o_ (low pass filter cut-off 1.6 Hz). Paired electrical stimulation was performed with bipolar platinum electrodes (wire diameter 25 μm, tip distance about 50–100 μm, pulse duration 0.1 ms, pulse interval 50 ms). They were positioned in the hilus (26), in stratum radiatum of the (pro)SUB, and in the white matter below layer VI of the TCx.

### Experimental protocols

#### Viability of slices

To exclude that drug effects were mimicked by loss of slice viability, we routinely determined amplitudes of averaged responses (*n* = 5) to the first response of paired stimulus-evoked FPs (every 20 s, stimulus intensities eliciting 80 or 100% of the maximal FP amplitude) in the beginning and at the end of the experiment. Experiments were accepted when the evoked signals did not change more than 25% between the beginning and end of an experiment.

#### Regionally optimized induction of seizure-like events

**Figure 1:**
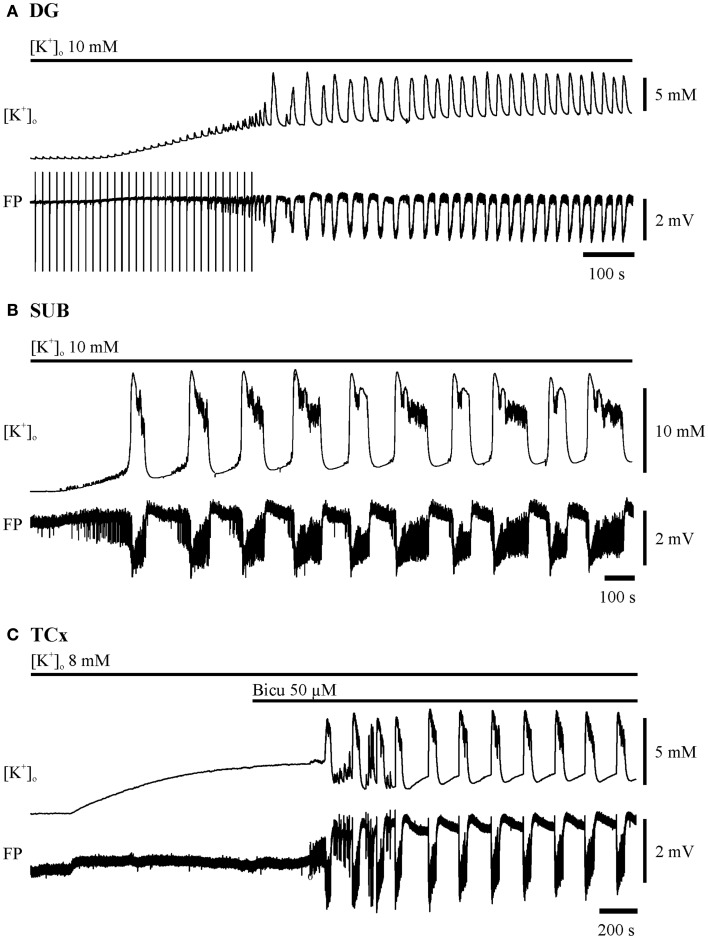
**Induction of seizure-like events in the dentate gyrus (DG), subiculum (SUB), and in deep layers of the temporal neocortex (TCx)**. The two traces depicted for each region display recordings of [K^+^]_o_ (top) and field potential (FP) bottom. Time and amplitude of signals are given by calibration bars on the right. Bars above each pair of traces mark the perfusion of the ictogenic buffer solution. **(A)** DG: hilar double pulse stimulation (pulse duration 0.1 ms, pulse interval 50 ms, stimulus intensity for pulses in the range of 80% of the maximal field potential amplitude, frequency 0.067 Hz). The stimulation was performed before and during elevation of [K^+^]_o_ to 10 mM, and had been set off when epileptiform discharges appeared independent of electrical pulses. **(B)** SUB: elevation of [K^+^]_o_ to 10 mM. **(C)** TCx: elevation of [K^+^]_o_ to 8 mM and addition of 50 μM Bicuculline when [K^+^]_o_ approximated the plateau of equilibration.

**Figure 2:**
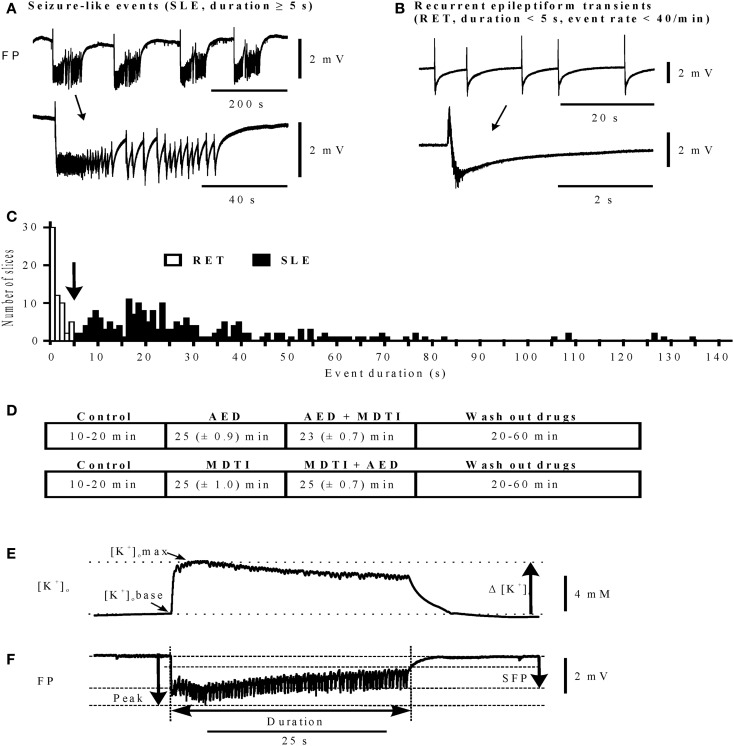
**Differentiation of epileptiform activities and determination of characteristic parameters**. **(A,B)** Traces of activity (top), single events marked by arrow are displayed on an expanded time scale (bottom), calibration bar for field potential amplitudes on the right. **(A)** Seizure-like events (SLEs); **(B)** recurrent epileptiform transients (RETs); **(C)** histogram of event durations for RETs and SLEs, bin width 1 s; **(D)** protocol sequences in main experiments: top (*n* = 120): control, AED, AED + MDTIs, and Wash out of drugs; bottom (*n* = 41): control, MDTIs, MDTIs + AED, and Wash out of drugs. For periods of Control and Wash out durations are given as Min and Max, and for the drug periods as Mean (±SEM). **(E)** Parameters determined for event-associated changes of extracellular potassium concentrations ([K^+^]_o_ in mM): [K^+^]_o_ base in the beginning of an event, [K^+^]_o_ max at the maximum value, Δ [K^+^]_o_ rise of [K^+^]_o_; **(F)** parameters determined for event-associated deviations of the field potential (FP): sfp (mV) maximum of the slow field potential amplitude, peak (mV) maximum of the peak amplitude, and duration (s) event duration (time from beginning of the event up to 2/3 recovery of the sfp deflection). **(E,F)** Calibration bar for [K^+^]_o_ and FP on the right. Abscissa in **(A,B,E,F)**: time in seconds (calibration bar below the FP traces).

In the DG, hilar stimulation and elevation of potassium concentration to 10–12 mM induced SLEs (26, 31) (Figure 1A). In the SUB elevation of potassium concentration to 10–12 mM could provoke SLEs (Figure 1B) while in the TCx 8 mM [K^+^] and 50 μM bicuculline–methiodide were sufficient. Bicuculline was added when [K^+^]_o_ approached the plateau of equilibration (see Figure 1C). SLEs lasted for more than 5 s (Figures 2A,C). Epileptiform events shorter than 5 s were termed recurrent epileptiform transients (RETs; Figures 2B,C). Slices in which only RETs were observed were not included in the analysis. The differentiation between SLEs and RETs was based on the histogram of event durations (Figure 2C) and on the relation between event duration and event rate (not shown). The distribution of values displayed a first minimum around 5 s and a second minimum around 15 s. However, only the first minimum was associated with a change in the event rate. Therefore, we decided to categorize SLEs by a minimal event duration of 5.1 s.

#### Treatment protocols

**Figure 3:**
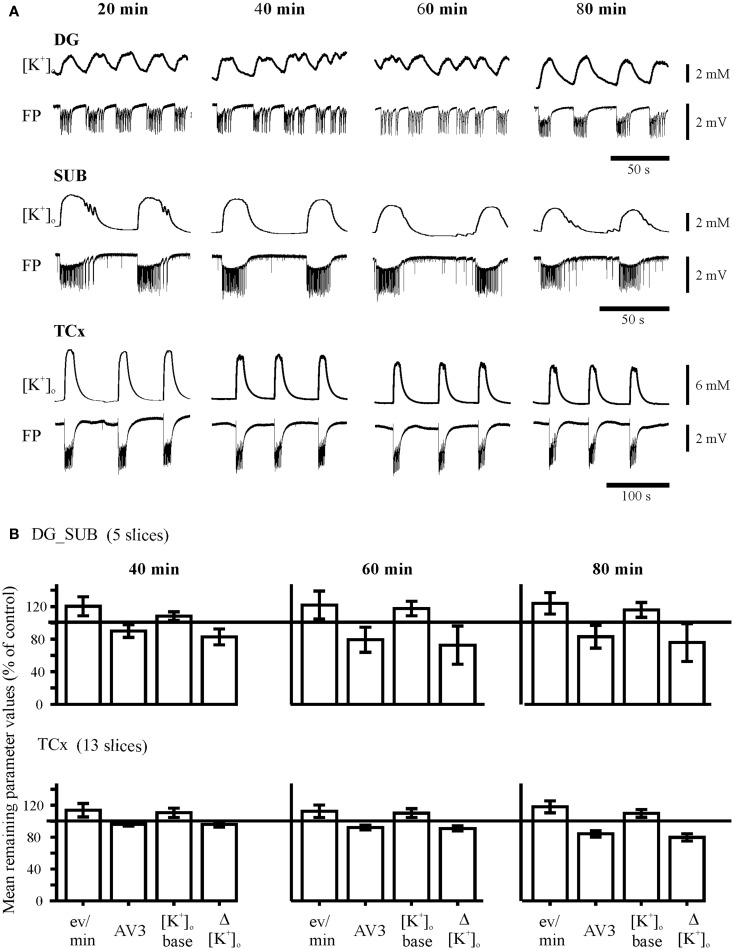
**Control experiments**. **(A)** Time-matched experiments without any drug perfusion in the dentate gyrus (DG), subiculum (SUB), and the temporal neocortex (TCx). The recordings of [K^+^]_o_ and field potential (FP) were taken from the end of each time interval, marked above traces. Calibration bars for amplitude and time are given on the right. **(B)** Time matched remaining parameter values (percent of control values). The remaining parameter values for event rate (eV/min), AV3 (averaged remaining normalized parameter values for sfp, peak, and duration), [K^+^]_o_ base, and ([K^+^]_o_ show variability or slight run down but no pattern change throughout time.

After induction of SLEs, slices were subjected to two different sequential protocols denoted as “AED” followed by “AED + MDTI” and as “MDTIs” followed by “MDTIs + AED” (Figure 2D). Moreover, a small number of control experiments have been performed in which slices were not perfused with drugs, denoted as “No drug”-experiments (Figure 3).

During the protocol “*AED*” (120 slices, 37 patients, Figure 2D, top), one of three AEDs CBZ, 50 μM, VPA, 1 mM, and PHT, 50 μM was applied for at least 20 min. Subsequently, during the protocol “*AED* + *MDTIs*,” the MDTIs PBN (400 μM) and/or VPM (40 μM) were added to the perfusion solution for another 20 min.

In order to test whether VPM and/or PBN may alter seizure-like activity independently of their interaction with potential AED transport, we also studied their effects on SLEs before the respective AEDs were added to the perfusate (Figure 2D, bottom). During this protocol, referred to as “*MDTIs*,” PBN, and/or VPM were applied first (41 slices, 15 patients) and then, in the protocol “*MDTIs* + *AED*,” one of the AEDs was added to the perfusion solution. The time schedule “MDTI” and “MDTI + AED” was similar to that for “AED” and “AED + MDTI.” During both protocols, the MDTIs VPM and PBN were simultaneously administered to 115 slices, PBN alone to 22 slices, and VPM alone to 22 slices.

The *“No drug”-protocol* (17 slices, 9 specimens, 7 patients) lasted 80 min without drug administration (Figure 3A). This permits to test for stability of induced activity, and to compare drug effects to a time-matched “No drug”-group (Figure S2 in Supplementary Material).

Drug effects were considered if at least a partial recovery of epileptiform activity was achieved. All substances were administered through the ictogenic perfusion medium.

### Immunohistochemistry of multidrug transport proteins

Expression patterns of multidrug transporters in specimens of drug resistant patients included in the present study have been already published (29). In brief, we used a modified glucose oxidase-diaminobenzidine (DAB) method (35). Tissue samples were fixed overnight (4% PFA) and 10 μm thin sections were cut in a cryostat (Leica, Jung CM 1800) and then incubated (24 h at 4°C) with diluted primary antibody [monoclonal antibodies: Pgp, JSB-1 antibody (1:50); MRP1, MRPr1 antibody (1:20); MRP2, M2III-6 antibody (1:50), Alexis Biochemicals; MRP5, M51-1 antibody (1:20) DCS/Signet, Hamburg, Germany]. The antibodies were diluted in normal goat serum (10%), Triton X-100 (0.3%), BSA, and 0.1 M PB (pH 7.4). Subsequently, slices were incubated for 1 h in biotinylated secondary antibody (1:100), washed in AB complex for 1 h, followed by DAB oxidation (ABC kit, Vector Labs Burlingame, CA, USA), and then counterstained with Vector Hematoxylin Nuclear Counterstain (Vector Labs).

Cell counting and quantification of immunohistochemistry data were carried out semi-automatically by using the software Kappa Image (Metreo Software, Kappa Optoelectronics) based on the method of West and Gundersen (36). The ratio of multidrug transporter expressing cells refers to the total cell number, determined in counterstained slices, and corrected following the method of Abercrombie (37). The ratio values were given in percent of the corresponding total cell number, averaged with respect to each transporter type, region, and cell type per patient.

### Data analysis and statistics

#### Initial analysis

Initial analysis was based on the categorization of effects in a given slice (persistence of SLE, transition of SLE to RET, or suppression of SLE). Quantification of drug effects followed previous protocols (26). Changes of [K^+^]_o_ were described (i) for the onset of events ([K^+^]_o_base), (ii) for the event-associated maximum of [K^+^]_o_ ([K^+^]_o_max), and (iii) for the event-associated rise of [K^+^]_o_ (Δ [K^+^]_o_ = [K^+^]_o_max − [K^+^]_o_base, Figure 2E). Deflections of the FP (Figure 2F) were characterized by their event rate (*n*/min), maximal slow FP amplitude [sfp (mV)], maximal peak amplitude [peak (mV)], considering the largest negative transient, and event duration [duration (s)]. Fluctuations with a size of <0.3 mV and a time interval of <3 ms were excluded from the analysis.

Moreover, all activity parameters were normalized with reference to the control value, determined after a stabilization period lasting 10–20 min, and given as remaining parameter values in a percentage of the control value. Three normalized parameter values (sfp, peak, and duration) were averaged as AV3 [see also Ref. (26)]. In order to prevent statistical overweight of event rate, normalized changes >150% were set to 150%.

In addition, *a patient oriented analysis* was performed, allowing correlation with clinical data. All pharmacological responses from more than one slice of the same specimen/patient were again categorized with respect to quality and heterogeneity. In order to relate some of our data to serum concentrations of clinically employed drugs with different pharmacokinetic properties and ranges of effectiveness, serum concentrations were normalized to the maximal therapeutic serum level for each AED [set by the clinical analysis, following available literature, i.e., Ref. (38)] and given as a percentage of the maximum level.

#### Statistical analysis

Group data of ratio variables are displayed as mean ± SEM throughout the manuscript. Data of nominal and ordinal variables are given as proportions of group members assigned to the response categories. As the Shapiro–Wilk tests indicated deviation from the normal distribution of values for some of the variables, comparisons within groups and between groups were performed using non-parametric tests (Wilcoxon, Friedman; Mann–Whitney *U*, Kruskal–Wallis-*H*). Proportional differences within and between groups were evaluated applying the McNemar and the Chi^2^-homogeneity test (Fisher’s exact test). All statistics were calculated using the PASW statistics 18 (SPSS, Chicago, IL, USA).

## Results

The present pharmacological study is based on experiments in 161 slices (35 hippocampal and 35 cortical specimens of 51 patients) in which SLEs could be induced (2038 with Table 1). First, we investigated pharmacosensitivity of SLEs to AEDs or MDTIs, and then to co-administration of AEDs and MDTIs.

**Table 1:** **Comparison of patient data with respect to *in vitro* sensitivity or resistance of SLEs to co-administration of one AED and probenecid or/and verapamil for 40 patients providing more than one slice to the analysis**.

Variables	Parameters or categories	Patients with sensitive tissue	Patients with resistant tissue	Statistics (*p*-value)
Frequency of seizures per month	Mean value	10.9 (26)	3.3 (13)	0.033
	±SEM	2.76	0.73	
Frequency of sGTCS per year	Mean value	7.37 (23)	7.46 (14)	0.586
	±SEM	4.42	3.19	
AED treatment at operation	CBZ or OXC	23.1% (26)	42.9% (14)	0.240
	LTG or GBT	38.5%	35.7%	
	LEV	30.8%	7.1%	
	TPM, ZNS, or PGL	7.7%	14.3%	
Brain-/serum level of AEDs^a^	Mean value (*N*)	1.5 (11)	1.05 (8)	0.322
	(±SEM)	0.21	0.31	
Pathology	No clear pathology	26.9% (26)	28.6% (14)	1.000
	Hippocampal pathology	42.3%	50.0%	
	Cortical pathology	19.2%	14.3%	
	Dual pathology	11.5%	7.1%	
Outcome (ILAE) 1–2 years after operation	Completely seizure free	58.3% (24)	85.8% (14)	0.564
	Auras only	12.5%	0.0%	
	One–three seizure days/a	12.5%	7.1%	
	Up to 50% reduction^a^	8.3%	0.0%	
	<50% reduction^a^	8.3%	7.1%	
MRP ne mean expression rate	Mean value	21.1% (8)	17.2% (7)	0.418
	±SEM	2.04	3.33	
MRP as mean expression rate	Mean value	32.9 (8)	25.1 (7)	0.247
	±SEM	4.07	4.81	
Pgp ne mean expression rate	Mean value	20.3 (8)	19.1 (7)	0.908
	±SEM	4.64	5.06	
Pgp as mean expression rate	Mean value	30.3 (8)	30.5 (7)	0.817
	±SEM	3.57	4.88	

*All patients experienced complex partial seizures*.

*Explanations: (*N*) = number of patients given once for the whole group, but always for reduced groups (data not determined, not available, or lost). “Sensitive” to co-administration means response in at least one slice of a patient; “resistance” denoted persistent SLEs in all slices. Corresponding mean number of slices per patient were 4.1 ± 0.38 and 3.1 ± 0.27 (*p* = 0.255)*.

*Categories of pathology: no clear pathology = Wyler degree 1 or 2 or discrete changes in TCx like moderate gliosis or single ectopic cells; hippocampal pathology = hippocampal sclerosis (Wyler degree 3 or 4), or tumor; cortical pathology = contusio, atrophy, dysplasia, tumor; dual pathology = hippocampal and cortical pathology*.

*^a^Categories of outcome: one to three seizure days per year; four seizure days per year up to 50% reduction of baseline seizure days; <50% reduction to 100% increase of baseline seizure days*.

*MRP ne, MRP as, Pgp ne, Pgp as: mean expression rate for neurons or astrocytes = number of immuno-positive cells given as percent of the total number of corresponding cells, determined and averaged for the investigated regions/specimens. The distribution of regions in the two patient groups is similar (*p* = 1.000 Fishers exact test)*.

*AED, anti-epileptic drugs; CBZ, carbamazepine; GBT, gabapentin; LEV, levetiracetam; LTG, lamotrigine; OXC, oxcarbazepine; PGB, pregabaline; PHT, phenytoinTPM, topiramate; VPA, valproic acid; ZNS, zonisamide.; SEM, standard error of the mean; sGTCS, secondary generalized tonic clonic seizures*.

*Statistics: comparisons between the two patient-groups were performed by the Mann–Whitney-*U*-test (numeric variables) and Fisher’s exact test (ordinal variables)*.

**p* = error probability*.

As shown in Figures 1 and 2A, SLEs were usually characterized by a large negative FP-shift of more than 5 s duration, associated with a rise in [K^+^]_o_. Event durations varied considerably (Figure 2C). In a given slice, SLEs recurred regularly after induction. In No drug experiments, the incidence of SLEs increased with time while duration and rises in [K^+^]_o_ declined with time (Figure 3B). Also, the value of AV3 became reduced. However, these changes were <20% in hippocampal slices and <10% in cortical slices. Spontaneous transition of SLEs to RETs was not observed in any of the 17 slices. SLEs were not *a priori* resistant (26) (here Figure S1 in Supplementary Material).

### Resistance of SLEs against carbamazepine, valproic acid, and phenytoin extends to cortical tissue slices

**Figure 4:**
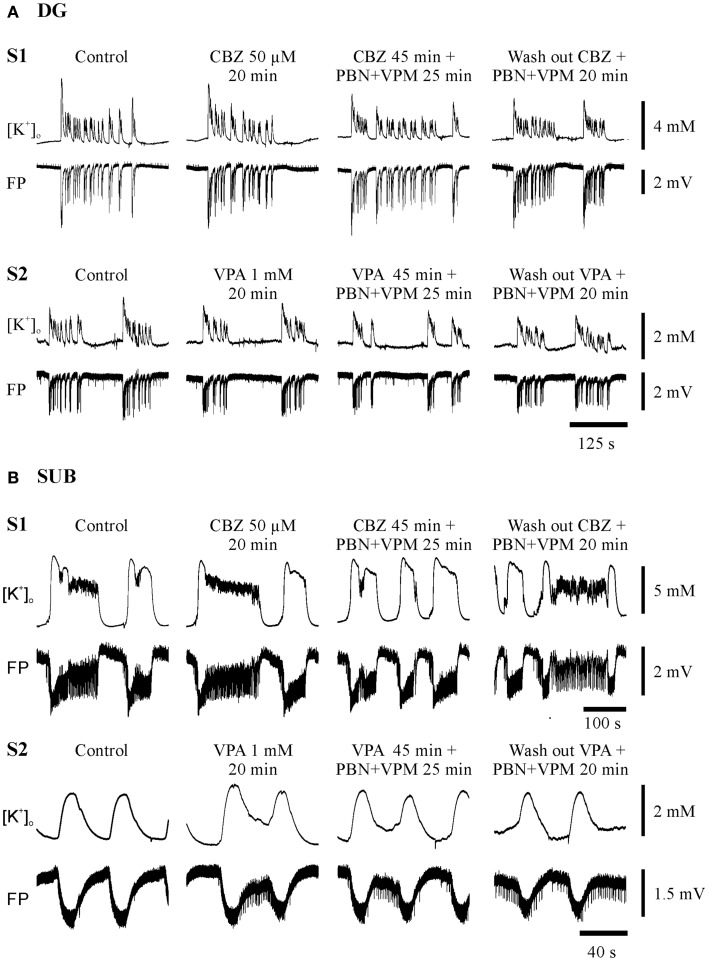
**Typical experiments in sister-slices from the same hippocampal specimen show persistence of SLE at the end of each protocol sequences (control, AED, AED + MDTIs, washout)**. **(A)** In the dentate gyrus (DG), **(B)** in the subiculum (SUB), S1 slice 1 with application of CBZ, S2 slice 2 with application of VPA for both regions. The drugs applied are described above the pairs of traces, which display [K^+^]_o_ (top), and field potential (FP) bottom. Amplitudes and time are given by calibration bars on the right.

**Figure 5:**
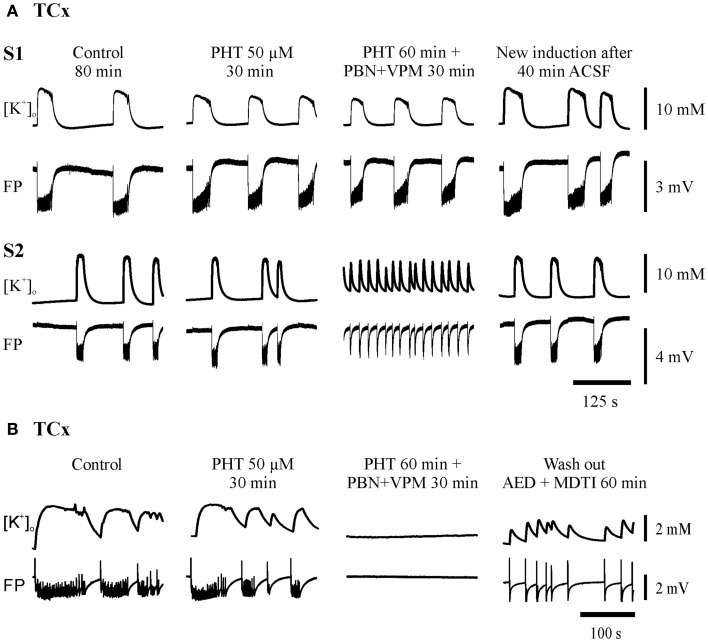
**Typical experiments in slices from TCx-specimens**. The traces were taken at the end of all subsequent protocol sequences (control, AED, AED + MDTIs, washout). **(A)** In sister-slices from the same specimen: S1 displaying resistance of SLEs against PHT and PHT + PBN + VPM; S2 illustrates resistance against PHT but transition of SLE to RET during co-administration (PHT + PBN + VPM); **(B)** in another specimen also displaying resistance of SLE against PHT but suppression of SLE during co-administration (PHT + PBN + VPM). The drugs applied are described above each pair of traces, which display [K^+^]_o_ (top) and field potential (FP) (bottom). Calibration bars for amplitude and time are given on the right.

Seizure-like events predominantly persisted in slices from hippocampal and temporal neocortical specimens. Figures 4 and 5 give examples of drug effects on induced SLEs in the DG, SUB, and TCx in sister slices from the same hippocampal or cortical specimen and show that SLEs persisted in presence of CBZ, VPA, and PHT.

**Figure 6:**
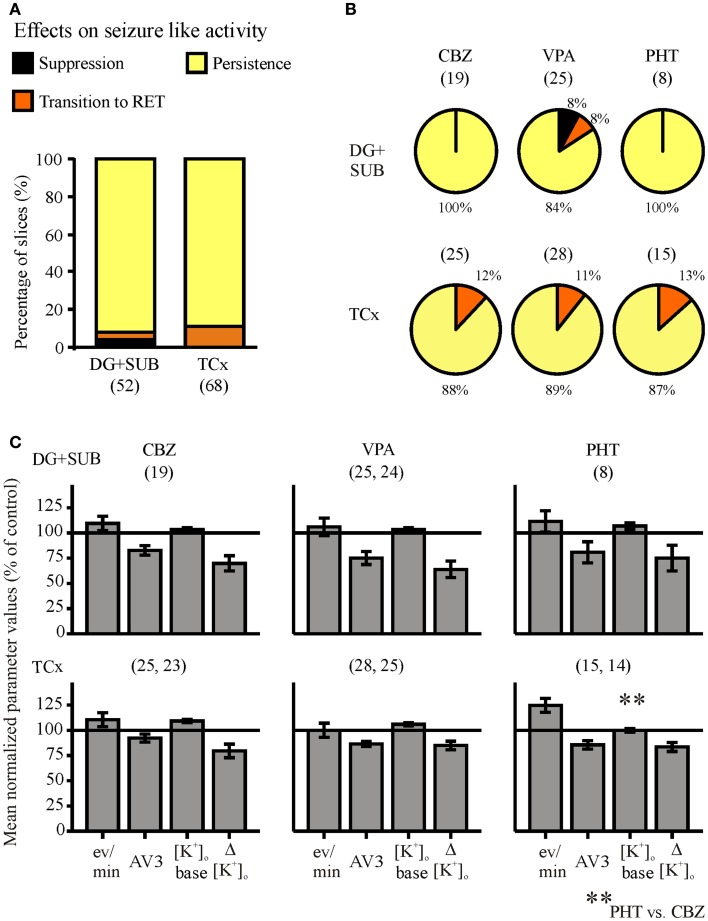
**Summarized effects of anti-epileptic drugs (AEDs) on seizure-like events**. Categories of drug effects, marked by different colors, are shown on top of **(A)**. **(A)** Proportional distribution of AED-effects in hippocampal and cortical slice-groups. **(B)** Effects of carbamazepine (CBZ), valproate (VPA), and phenytoin (PHT) in hippocampal (top) and neocortical slices (bottom). **(C)** Corresponding averaged remaining normalized parameter values for event rate (eV/min), AV3 (average of normalized values for sfp, peak, and duration), [K^+^]_o_ base, and ([K^+^]_o_. Ordinates: **(A)** percentage of slices; **(C)** means ± SEM for remaining percentages of control values. Signs for significant differences between ranges of values for PHT vs. CBZ **p ≤ 0.01. Numbers in parenthesis give number of slices in sub-samples **(A,B)**, or number of slices analyzed **(C)**. The second numbers in parenthesis of **(C)** display the number of slices with reliable measurements of [K^+^]_o._

The synopsis of all AED drug experiments (Figure 6) revealed that initial SLEs (120 slices) persisted in 90% of slices, were replaced by RETs in 8.4%, and suppressed in only 1.6%, in spite of high AED-concentrations in the perfusion solution. Regarding the different specimens, AEDs were similarly effective in modifying SLEs in hippocampal and TCx slices (Figure 6A).

With respect to the three AEDs tested (Figure 6B), we found in slices from hippocampal and cortical specimens that effects of CBZ, VPA, and PHT were not significantly different, suggesting that the efficiency of the three AEDs in the chosen concentration range is similar. Studies of drug effects on seizure parameters (Figure 6C) also revealed that there were no significant differences between the different drugs, except for the baseline [K^+^]_o_ in slices from cortical specimens, which differed between CBZ and PHT.

### Seizure-like activity also persists in the presence of drug transport inhibitors

**Figure 7:**
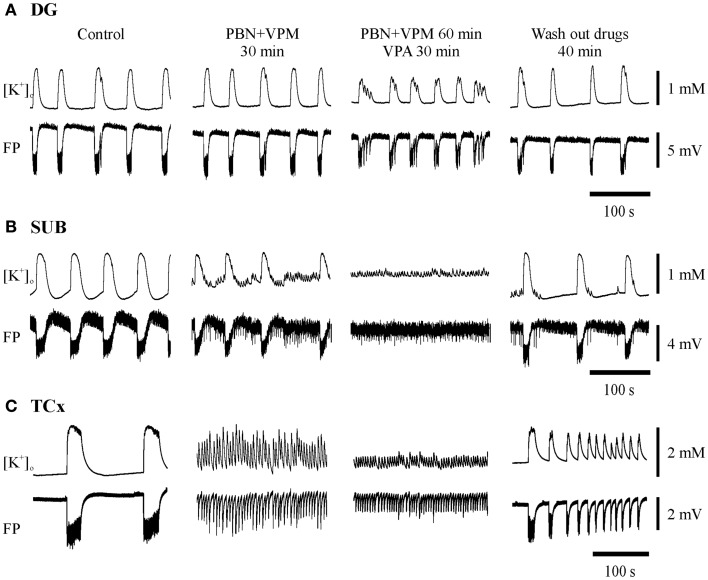
**Typical experiments with administration of probenecid (PBN) and verapamil (VPM) followed by addition of an AED in the three regions investigated**. Substances applied are described above the pairs of traces displaying [K^+^]_o_ (top) and FP (bottom). Traces were taken at the end of each protocol-sequence. **(A,B)** Synchronous recordings from one hippocampal specimen in DG and SUB display resistance of SLE against PBN + VPM in both regions while co-administration with VPA resulted in persistence of SLEs in the DG and in replacement of SLEs by RETs in the SUB; **(C)** recordings from a cortical specimen of another patient show transition to RET already after application of PBN + VPM, thereby indicating seizure modifying effects of MDTIs, which become more pronounced after co-administration with VPA. **(A,C)** Amplitudes and time are given by calibration bars on the right.

**Figure 8:**
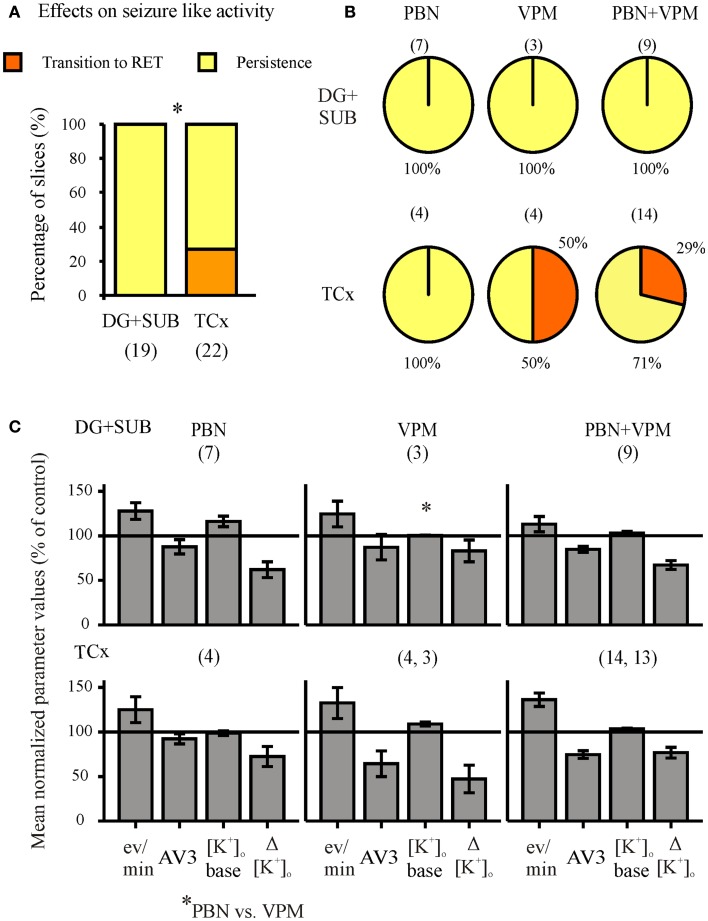
**Summarized effects of unspecific inhibitors of P-glycoprotein (Pgp) and multi-drug resistance-associated proteins (MRPs)**. Categories of drug effects, marked by different colors, are shown on top of **(A)**. **(A)** Proportional distribution of effects after administration of MDTIs in hippocampal and cortical slice-groups. **(B)** Circles representing the distribution of effects by probenecid (PBN), verapamil (VPM), and both PBN and VPM in hippocampal (top) and cortical slice-groups (bottom). **(C)** Corresponding averaged remaining normalized parameter values for event rate, AV3 (average of normalized values for sfp, peak, and duration), [K^+^]_o_ base, and ([K^+^]_o_. Ordinates: **(A)** percentage of slices; **(C)** means ± SEM for percentages of control values. Signs for significant differences between proportional distributions *p ≤ 0.05, or ranges of values for PBN vs. PBN + VPM *p ≤ 0.05. Numbers in parenthesis give number of slices in sub-samples **(A,B)**. The numbers in **(B)** also apply to **(C)**.

When the MDTIs VPM and/or PBN were applied first (Figure 7, second column of traces; Figure 8, summary of 41 slices), the proportion of slices with continuing SLEs amounted to 85.4%. SLEs were never suppressed but could be transformed to RETs. Regional differences in sensitivity to MDTIs were observed. In hippocampal slices (*n* = 19) SLEs persisted in 100% of slices while in cortical slices (*n* = 22) SLEs persisted only in 73% (*p* = 0.023, Fisher’s exact test), indicating that induced activity in cortical slices was more sensitive to MDTIs than in hippocampal slices (Figure 8A). Some slices were investigated for singular effects of PBN (*n* = 11) or VPM (*n* = 7). There were no proportional differences between effects of PBN, VPM, and PBN plus VPM in hippocampal as well as cortical slices (Figure 8B). Nevertheless, it should be noted that in four temporal cortex slices in which VPM was applied alone (Figure 8B, bottom), this drug led to transition of SLE into RETs in two slices, an effect that was not seen with PBN. Similarly, seizure parameters in temporal cortex slices (Figure 8C) showed that effects of VPM were conspicuously larger than for PBN. However, the difference could not be proven significant, due to the small number of slices tested.

### Co-administration of AEDs and MDTIs increases responses of slices

**Figure 9:**
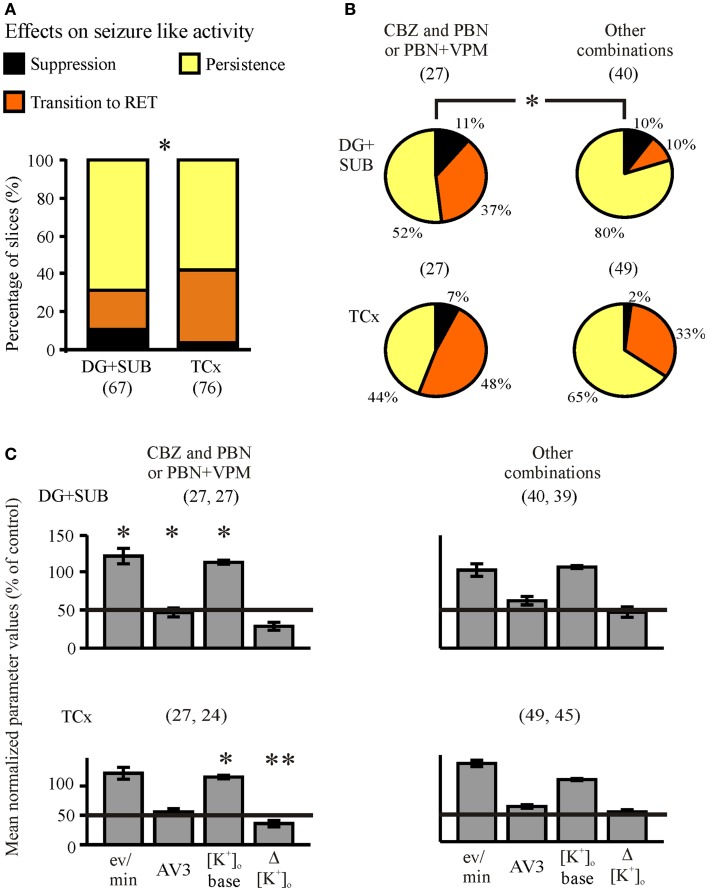
**Summarized effects of one AED and MDTIs (co-administration)**. Categories of drug effects, marked by different colors, are shown on top of **(A)**. **(A)** Proportions of slices showing different effects of co-administration in hippocampal and cortical slice-groups. **(B)** Proportional distributions of effects by CBZ and PBN-containing solutions (CBZ + PBN, CBZ + PBN + VPM) and by other combinations (CBZ + VPM, VPA + PBN, VPA + VPM, VPA + PBN + VPM, and PHT + PBN + VPM), given in circles for hippocampal (top) and cortical slice-groups (bottom). **(C)** Corresponding averaged remaining normalized parameter values for event rate (eV/min), AV3 (average of normalized values for sfp, peak, and duration), [K^+^]_o_ base, and ([K^+^]_o_. Ordinates: **(A)** percentage of slices; **(C)** means ± SEM for remaining percentages of control values. Signs for significant differences between proportional distributions of effects or between ranges of values in separate groups (treatment or regions) *p ≤ 0.05, **p ≤ 0.01. Numbers in parenthesis give number of slices in sub-samples **(A,B)** or number of slices analyzed **(C)**. The second number in parenthesis of **(C)** displays the corresponding number of slices with reliable measurements of [K^+^]_o._

In order to find out whether co-administration of anti-seizure drugs and MDT-inhibitors is able to reverse resistance to standard AEDs, we added PBN and/or VPM to the perfusion solution already containing one AED (AED + MDTIs) (Figures 4 and 5, third column of traces). Alternatively, one AED was added to the perfusion solution already containing MDTIs (MDTIs + AED) (Figure 7, third column of traces). As there were no differences of co-administration effects on SLEs resistant against AEDs or MDTIs (*n* = 143, *p* = 0.234, Fisher’s exact test), the subsamples AED + MDTI and MDTI + AED were pooled. Together, during combined treatment with an AED and MDTIs, SLEs persisted in 63% of slices, became converted into RETs in 30%, and suppressed in 7%. Thus, co-application of an AED and MDTIs resulted more frequently in transition to RET compared to slices treated with AEDs or MDTIs alone (*p* < 0.001, McNemar–Bowker test). Different from slices of cortical specimens, SLEs in hippocampal slices continued more often (69 vs. 58%), were more often suppressed (10.4 vs. 3.9%), and less often transformed to RET (21 vs. 38%; Figure 9A, *p* = 0.042).

Concerning the combinations of AEDs and MDTIs administered (Figure 9B), we found that SLEs were more often sensitive to CBZ + PBN or CBZ + PBN + VPM (*n* = 54) than to all other combined applications (*n* = 89, *p* = 0.017, Fishers exact test). Although this was numerically true in slices from hippocampal and cortical specimens, the error probability reached significance (*p* = 0.022) in hippocampal specimens only. Out of the remaining normalized parameter values event rate, AV3 and [K^+^]_o_ base significantly differed between the combinations in the hippocampus, while [K^+^]_o_ base and Δ[K^+^]_o_ differed in the cortex (Figure 9C). Taken together, co-administration of one AED and MDTIs modifies appearance of SLEs by transition to RETs but rarely by suppression.

### Co-administration experiments reveal homogeneous and heterogeneous responses in slices of the same patient

When studying the effects of CBZ in the DG of hippocampal slices from the same patient (26), we noted that the substance failed to suppress SLEs in one slice but was effective in another. This may indicate differences of drug sensitivity throughout the resected tissue, prompting investigation of multiple slices from each patient.

**Figure 10:**
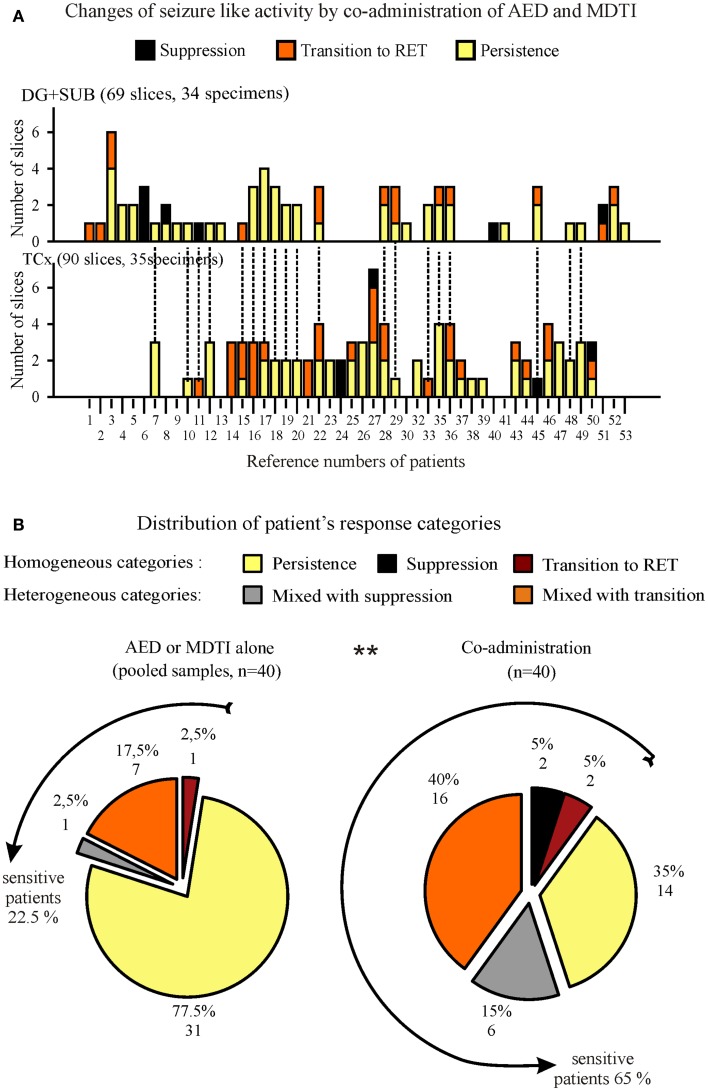
**Summarized responses to co-administration of AED and MDTIs in specimens and patients**. **(A)** Effects in slices from hippocampal (top) and cortical (bottom) specimens, ordered by patient number for determination of the patient’s response category. Patients providing both, hippocampal and cortical specimens are marked by dotted lines on top of columns in **(B)**. **(B)** Proportional distribution of different response categories for patients with at least two slices in the analysis. In order to evaluate the changes by co-administration, we present two circles, one for responses to AED or MDTIs alone (pooled sample, left) and another one for responses to co-administration (right). The lines around circles indicate subgroups of patients who were “sensitive” to the respective treatment in at least one slice. Numbers in parenthesis denote the number of slices/specimens **(A)** or the number of patients **(B)**.

One to seven slices from one specimen could be investigated for responses of SLEs to co-administration of AEDs and MDTIs (Figure 10A). In specimens with more than one slice (Figure S4 in Supplementary Material), we observed different effects in 52.6% of 19 hippocampal specimens and in 42.9% of 28 cortical specimens. Consequently, categorization of the patient’s *in vitro* drug response also needs consideration of heterogeneity of effects in two or more slices of the same patient.

In summary of both specimens (Figure 10B), 40 patients provided between 2 and 7 slices to the categorization procedure. In Figure 10B (left), responses to AED or MDTIs alone were summarized. Pharmacoresistance was homogenous in 77.5% of patients. In the remaining 22.5% of patients, sensitivity to AED or MDTIs was found in at least one slice. In Figure 10B (right), the combined effects of AEDs and MDTIs were summarized. The graph shows a significantly larger proportion of patient material in which we observed sensitivity (65%). Compared to treatment with either AEDs or MDTIs alone, we noted a strong increase in the sensitivity to co-administration of one AED and MDTIs (*p* = 0.001, McNemar).

### Relations of *in vitro* response categories to patient data

Patients’ with at least two slices in the analysis were compared between *in vitro* “sensitive” and “resistant” groups concerning co-administration of an AED and MDTIs as displayed in Table 1.

We also tested for age at resection, onset of epilepsy, gender, and site of resection and found no significant differences between the two groups (*p* = 0.314; 0.100, 0.739, 0.510). The two patient groups only differ with respect to the distribution of *in vivo* seizure frequencies (high for sensitive patients, low for resistant patients) but not between their quotients of brain tissue- to serum-concentrations of AEDs. Besides, we recognized that local tissue concentrations of AEDs or their metabolites (in case of CBZ or OXC) are positively correlated to corresponding serum concentrations in the hippocampus (Pearson’s *r* = 0.764, *n* = 18, *p* < 0,001) but not in the neocortex (*r* = 0.253, *N* = 15, *p* = 0.362). Also, the local expression rates of MRPs and Pgp determined for neurons and astrocytes in corresponding subgroups do not differ (Table 1). These findings put doubt on a relevant role of Pgp and MRPs in parenchymal drug resistance.

To find out, whether specimen-specific variations of patient data may differ, we performed a separate analysis for both specimens and display a corresponding table for TCx-specimens in the Supplementary Material (Table S1 in Supplementary Material). The error probability of 0.046 for the difference between brain/serum concentrations of AEDs between resistant and sensitive tissue in TCx-specimens was not accepted as “significant” (SPSS 18) because of the small sample sizes and the large spread of values in both groups.

Compared with the patient groups providing TCx-specimens (sensitive vs. resistant) corresponding subgroups of hippocampal specimens differ concerning age of onset (8.5 ± 2.92 years, *n* = 6 vs. 22.0 ± 3.61 years, *n* = 11) and gender [sensitive patients are mainly male (75%) resistant patients mainly female (82%, *p* = 0.024 Fisher’s exact test)]. For subgroups in both specimens (TCx, Hippocampus), there is no difference with respect to pathologies (TCx *p* = 0.124, Hippocampus *p* = 0.500 for Wyler graduation), resistance, or AED-treatment at operation.

## Discussion

In the present study, we investigated effects of AEDs and inhibitors of Pgp and MRPs on artificially induced recurrent SLEs in slices from hippocampal and cortical specimens of drug resistant patients.

### AED-resistance of patients is preserved in the resected tissue

The vast majority of slices retained SLEs when CBZ, VPA, or PHT was applied. In a previous study of the DG, we described that epileptiform activity induced in slices of drug resistant patients remained predominantly resistant to CBZ (26). Our present findings on seizure-like activity support the previous statement and we now show that this applies also to VPA or PHT. Additionally, the results indicate that not only the DG but also the SUB and TCx of surgically resected tissue are frequently characterized by pharmacoresistance of SLEs. Considering that in certain patients the blood brain barrier is leaky (39–41), and that drug concentrations in the tissue are close to plasma levels or even exceed those (42), we used high AED-concentrations in the tissue experiments. Despite such high concentrations, SLEs were resistant.

The present results are in line with reports on effects of AEDs on epileptiform activity in human cortical slices, focused on short recurrent discharges induced by lowering [Mg^2+^] in the perfusion medium. In a study by Oby and colleagues, application of 50 μM CBZ or PHT resulted in a slight modification of the repetition rate and a decline of the integrated amplitude of short recurrent epileptiform discharges (43). Levetiracetam in human temporal cortex slices did not affect spontaneous sharp waves or Mg^2+^-free induced epileptiform FPs (44). The agreement of results from several studies using different induction protocols suggests that pharmacological investigations aiming at detection of new suitable drugs for drug resistant patients are also possible in centers where only neocortical tissue is available.

### Verapamil and probenecid partially modify induced epileptiform activity in cortical slices

Verapamil and PBN were used as broad spectrum drug transport inhibitors. However, these agents also have unspecific anticonvulsant effects. Indeed, VPM and PBN, administered in the absence of AEDs, led to transformation of SLEs into RETs in 30% of TCx slices, which was never observed in the DG or SUB. This effect might be related to direct anticonvulsant effects.

Verapamil, known as an organic L-type calcium channel antagonist, was shown to exert anticonvulsant effects in a number of experimental models of epilepsy (45–49). Even in human tissue, VPM reduced spontaneous FP transients and low Mg^2+^ and bicuculline-induced epileptiform activity (50, 51).

Less is known about anticonvulsant effects of PBN. PBN might affect release of ATP and (conversion to) adenosine (52) as well as regulatory processes within a complex metabolic chain providing for neuroprotective effects of the NMDA-receptor inhibitor kynurenic acid and also the neurotoxic quinolinic acid (53, 54). Substances such as adenosine (55–59), taurine (60, 61), and kynurenic acid (54) are known to exert anticonvulsant effects. More recently, it was suggested that PBN blocks pannexin hemi channels (62), which might contribute to seizure activity when opened (63). Thus, our finding that PBN and VPM administered before application of CBZ, VPA, or PHT can convert SLEs into RETs suggests that any of those agents or their combination support the effect of AEDs and therefore might indicate a rational basis for multi drug treatment in some instances.

### Is there evidence for a contribution of Pgp or MRPs to drug resistance?

Clinical data exist, which show that VPM can sometimes be used as an additive drug in the treatment of refractory epilepsy (28), in severe myoclonic epilepsy in infancy (64), and in the treatment of pharmacoresistant status epilepticus (65–67).

In the present study, co-administration of one AED and MDTIs in slices resulted more often in a transition of SLEs to RETs than administration of AEDs or MDTIs alone. However, full suppression of SLEs suggesting reversal of drug resistance was rarely observed. This would be in agreement with a previous report of Rivers and colleagues who could not assess reversion of the drug resistance phenotype and possible interactions with ABC-drug transporters for any of the AEDs used in our study (68).

We showed that CBZ co-applied with PBN− or PBN + VPM containing solutions was more effective than other combinations, particularly in the hippocampus. The finding that in the hippocampus weak or no effects were noted in presence of AEDs or MDTIs alone but strong effects during co-administration of CBZ and MDTIs might be a sign for interaction of metabolites of CBZ with MRPs in the hippocampus. However, our previous results on slices of a subgroup of similar patients showed that efflux pump function is preserved in both specimens (57% of temporal cortex slices and 60% of hippocampal slices). Moreover, corresponding sub-samples of slices did not differ with respect to expression of MDTs in the TCx and hippocampus (29).

We further calculated tissue-serum quotients of AED-levels in a subset of patients and found that they were only rarely and not homogeneously low. Although there are no data about the unbound fraction of AEDs in the present study, the value for total levels of AEDs in the tissue told us that AEDs reached the brain tissue in both subgroups, patients with *in vitro* sensitive as well as resistant tissue, at least in the hippocampus.

Provided that PHT is a substrate of Pgp but not VPA (69–71), blocking the transport might augment effects of PHT. However, a selective augmentation for PHT and MDTIs was not observed. Metabolites of CBZ with anticonvulsant or convulsant properties [Ref. (72) for a convulsant metabolite of CBZ] may be subject to drug efflux pump transport (73, 74). Therefore, it is of interest that we detected more frequently and stronger effects of CBZ when co-administered with PBN or PBN and VPM than for VPA and PHT in corresponding experimental conditions. In contrast, Luna-Tortos and colleagues reported that there was no PBN- or MK571-inhibitable transport of CBZ, VPA, and PHT in experiments on cell lines transfected with human MRPs (75).

Taken together, it seems to be less likely that our results on modification of SLEs can be ascribed to increased drug concentration at cellular targets of AEDs but rather are the consequence of combined drug effects at several anti-seizure targets.

### Heterogeneity of *in vitro* drug effects

Here, we show for the first time that slices of the same patient display different responses of SLEs to co-administration of one AED and MDTIs. This might be caused by different consequences of surgical handling and slicing in the laboratory as well as by different pathological changes in individual slices of the same specimen. To clarify the reasons for heterogeneity of pharmacological responses in slices of the same specimen a new study of differential morphological, biochemical, and immunological properties in “responding” and “non-responding” slices is ongoing. When responses of hippocampal and cortical specimens were categorized for treatment with AED or MDTIs, we noted mixed effects (resistance and pattern change) in 19% of patients. Such mixed effects increased to 54% during co-administration of one AED and MDTIs, and homogeneous suppression also increased from 0 to 7% of 41 patients. Nevertheless, the finding that there were no differences between the response categories regarding MRP- and Pgp local expression rates or tissue/serum quotients of AED-concentrations suggest a rather marginal contribution of MDTs to drug resistance in tissue without a functional blood brain barrier.

### Limitations

1. We did not observe spontaneous SLEs in human tissue slices and needed induction of seizure-like activity by modification of the electrolyte supplying the slices. The fact that spontaneous seizures were never detected presumably results from interruption of essential connectivity by surgery and preparation of slices. Even if the tissue would be capable to generate SLEs spontaneously, the low incidence of seizures in TLE patients would make it unlikely that we observed spontaneous seizures during the possible observation time.
2. Our study does not address the mechanisms underlying different vulnerability of hippocampal and neocortical tissue to modifications in potassium concentration. With respect to the requirement of bicuculline for induction of SLEs in slices of cortical specimens it might be of advantage to synchronously investigate changes of inter-neurons concerning morphology and molecular biology of their afferent connections and synaptic transmission to target cells.
3. It is presently not known whether resistance of induced SLEs in slices reflects resistance of spontaneous seizures of patients and if potential mechanisms of multidrug resistance found in this model system can be translated to the clinical setting of refractory epilepsy. None of the available models of pharmacoresistance so far is clinically validated. There are no drugs available, which are more effective in such models than the clinically employed AEDs. This is also true for the induced SLEs in this study. However, when potential seizure controlling drugs are effective *ex vivo* in human tissue, this might encourage further development and eventually testing in pharmacoresistant patients.
4. The non-selectivity of PBN and VPM with respect to inhibition of drug transport provides no clear argument against the design of our co-administration experiments. We found similar effects of adding MK 571 or PBN to VPA and VPM in the same slice (Figure S3 in Supplementary Material). Additionally, our findings that VPM and PBN altered drug efflux pump function in slices (29) support the use of VPM and PBN as drug transport inhibitors.

## Conclusion

The results of the present study indicate that SLEs in resected human temporal cortex and hippocampus are predominantly resistant to CBZ, VPA, and PHT as well as to drug transport inhibitors like VPM and PBN.

Complete suppression of seizure-like activity was rarely observed after co-administration of an AED with PBN and VPM, suggesting that drug resistance in slices is not reversed by inhibition of drug transport with PBN and VPM. This suggestion is supported by the fact that patients with *in vitro* sensitive tissue do not differ from patients with *in vitro* resistant tissue concerning expression rates of Pgp and MRPs or quotients of tissue/serum levels of anticonvulsants.

Co-administration of an AED with PBN and VPM *in vitro* increased the efficiency in modifying induced seizures (SLEs), rather by adding several anticonvulsant effects than by interaction with drug transport.

The heterogeneity of drug effects in different slices of the same patient remains to be elucidated.

Provided that the resistance of induced SLEs as well as the heterogeneity of drug effects reflect rather disease dependent than methodological consequences, our findings suggest that human tissue slices can be used for the evaluation of new drugs for patients with drug refractory epilepsy, i.e., by performing experimental multi-center studies.

## Conflict of Interest Statement

The authors declare that the research was conducted in the absence of any commercial or financial relationships that could be construed as a potential conflict of interest.

## Supplementary Material

The Supplementary Material for this article can be found online at http://www.frontiersin.org/Journal/10.3389/fneur.2015.00030/abstract

## Abbreviations

AEDs, anti-epileptic drugs; CBZ, carbamazepine; DG, dentate gyrus; FP, extracellular field potential; [K^+^]_o_, extracellular potassium concentration; MDTs, multidrug transporters, MDTIs, multidrug transporter inhibitors; MRP, multidrug resistance-associated protein; PBN, probenecid; Pgp, P-glycoprotein; PHT, phenytoin; RETs, recurrent epileptiform transients; SLEs, seizure-like events; SUB, subiculum; TCx, temporal neocortex; TLE, temporal lobe epilepsy; VPA, valproate; VPM, verapamil.
