# Supplementary material for: Drug Resistance in Cortical and Hippocampal Slices from Resected Tissue of Epilepsy Patients: No Significant Impact of P-Glycoprotein and Multidrug Resistance-Associated Proteins
Source: Front Neurol. 2015 Feb 18;6:30. doi: 10.3389/fneur.2015.00030 (PMC4332373; doi:10.3389/fneur.2015.00030)
Supplement: Supplementary file 1 [file data_sheet_1.docx]

**Supplementary Table 1:** Comparison of patient data for TCx-specimens with respect to *in vitro* sensitivity or resistance of SLEs to co-administration of one AED and probenecid or/and verapamil for 28 patients providing more than one TCx-slice to the analysis All patients suffered from Temporal lobe epilepsy and experienced complex partial seizures.

**Explanations:** (N) = number of patients (reduced when data were not determined, not available, or lost). “Sensitive” to co-administration means response in at least one slice of a patient; “resistance” denoted persistent SLEs in all slices. Corresponding mean number of slices per patient were 3.25 ± 0.31 and 2.6 ± 0.193 (p = 0.096). **Categories of TCx-pathology:** discreet = no pathology; moderate signs = moderate gliosis or single ectopic neurons; Tumor = Cavernoma, Ganglioglioma, or Astrocytoma; FCD = focal cortical dysplasia, here the one case has no special classification; Lesion, Atrophy; **Categories of outcome *:** four seizure days per year up to 50% reduction of baseline seizure days; less than 50% reduction to 100% increase of baseline seizure days; **MRP ne, MRP as, Pgp ne, Pgp as:** mean expression rate for neurons or astrocytes = number of immuno-positive cells given as percent of the total number of corresponding cells, determined and averaged for the investigated specimens.. **Abbreviations:** AED anti-epileptic drugs: CBZ carbamazepine, GBT gabapentin, LEV levetiracetam, LTG lamotrigine, OXC oxcarbazepine, PGB pregabaline, PHT phenytoin, TPM topiramate, VPA valproic acid, ZNS zonisamide; SEM: standard error of the mean; sGTCS: secondary generalized tonic clonic seizures; **Statistics:** comparisons between the two patient-groups were performed by the Mann-Whitney-U-test (numeric variables) and Fisher’s exact test (ordinal variables). p = error probability. The error probability of 0.046 for the difference between brain/serum concentrations of AEDs between resistant and sensitive tissue in TCx-specimens was not accepted as significant because of the small sample sizes and the large spread of values in both groups.

| **Variables** | **Parameters or categories** | **Patients with sensitive tissue** | **Patients with resistant tissue** | **Statistics (p -value)** |
| --- | --- | --- | --- | --- |
| **Frequency of seizures per month** | mean value  ± SEM | 13.6 (16)  4.06 | 15.0 (12)  12.28 | **0.024** |
| **Frequency of sGTCS per year** | mean value  ± SEM | 8.6 (15)  6.64 | 3.2 (12)  1.54 | 0.780 |
| **AED treatment**  **at operation** | CBZ or OXC  LTG or GBT  LEV  TPM, ZNS, or PGL | 25.0 % (16)  31.3 %  43.8 %  0.0 % | 58.3 % (12)  25.0 %  8.3 %  8.3 % | 0.075 |
| **brain-/serum level of AEDs*** | mean value (N)  (± SEM) | 1.6 (7)  0.32 | 0.64 (6)  0.26 | 0.046 |
| **TCx-Pathology** | discreet  moderate signs  Tumor  FCD  Lesion, Atrophy | 56.3 % (16)  18.8 %  18.8 %  0.0 %  6.3 % | 33.3 % (12)  50.0 %  0.0 %  8.3 %  8.3 % | 0.124 |
| **Outcome**  **(ILAE)**  1-2 years  after operation | completely seizure free  auras only  one - three seizure days/a  up to 50 % reduction*  less than 50 % reduction* | 60.0 % (15)  6.7 %  6.7 %  13.3 %  13.3 % | 83.3 % (12)  0.0 %  8.3 %  0.0 %  8.3 % | 0.797 |
| **MRP ne** mean expression rate | mean value  ± SEM | 25.5% ( 4)  3.64 | 24.7 % (4)  2.32 | 0.773 |
| **MRP as** mean expression rate | mean value  ± SEM | 30.5 ( 4)  0.75 | 21.5 (4)  4.07 | 0.083 |
| **Pgp ne** mean expression rate | mean value  ± SEM | 15.9 (4)  5.23 | 27.9 (4)  5.06 | 0.386 |
| **Pgp as** mean expression rate | mean value  ± SEM | 33.4 ( 4)  1.48 | 26.2 (4)  4.24 | 0.149 |
