# Supplementary figures and images for: Drug Resistance in Cortical and Hippocampal Slices from Resected Tissue of Epilepsy Patients: No Significant Impact of P-Glycoprotein and Multidrug Resistance-Associated Proteins
Source: Front Neurol. 2015 Feb 18;6:30. doi: 10.3389/fneur.2015.00030 (PMC4332373; doi:10.3389/fneur.2015.00030)

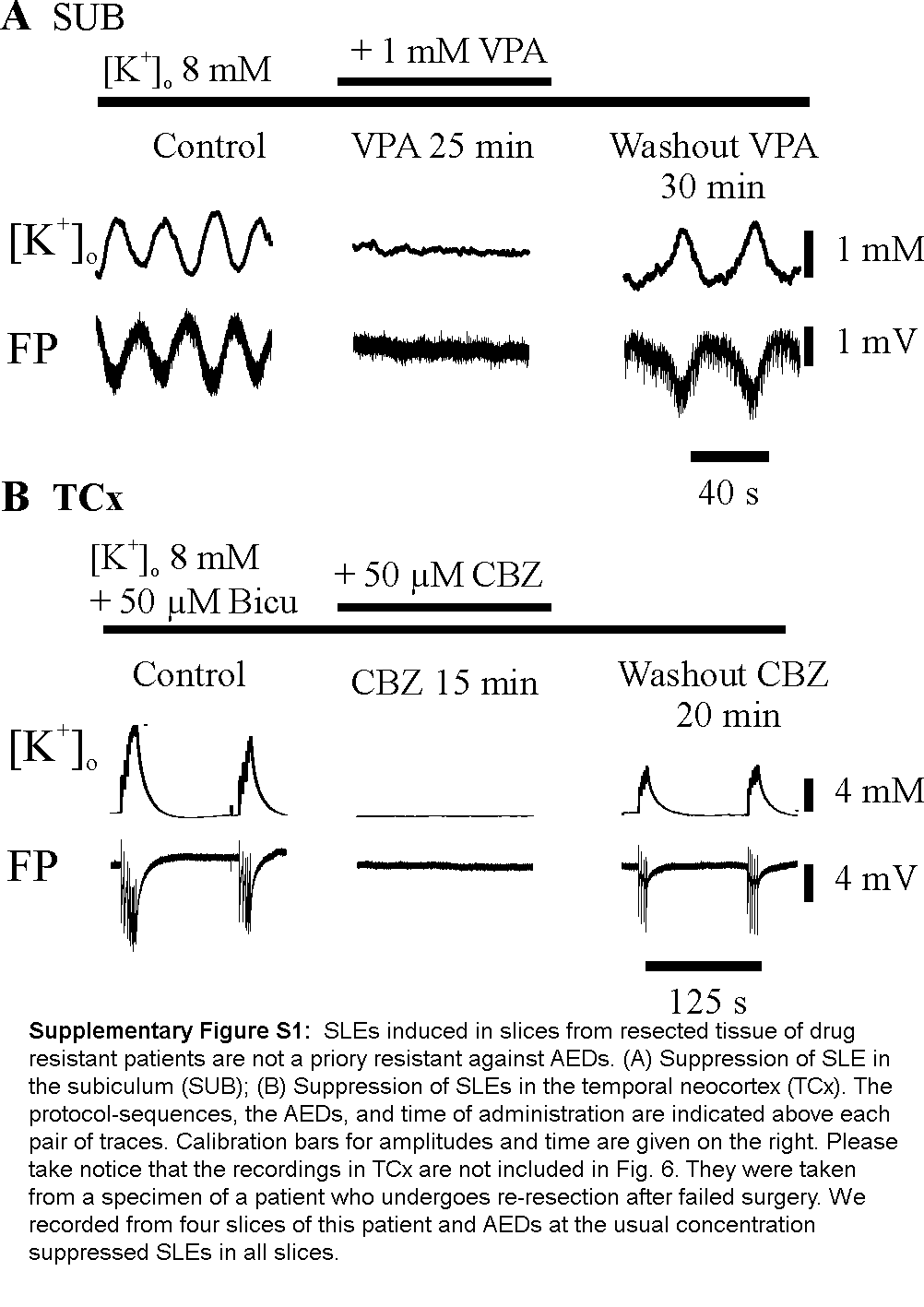

Supplement: Supplementary file 2 [file image_1.tif]

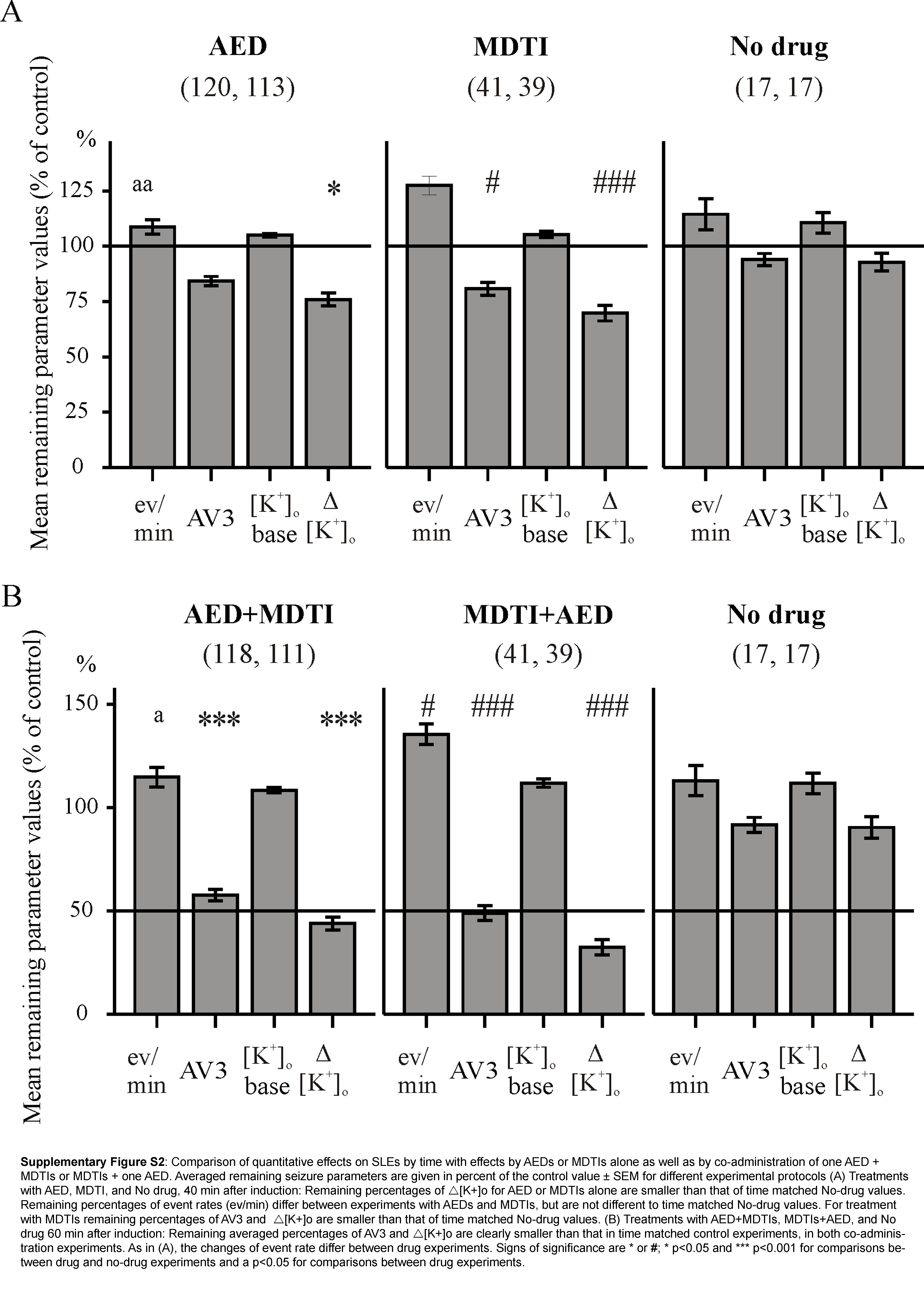

Supplement: Supplementary file 3 [file image_2.tif]

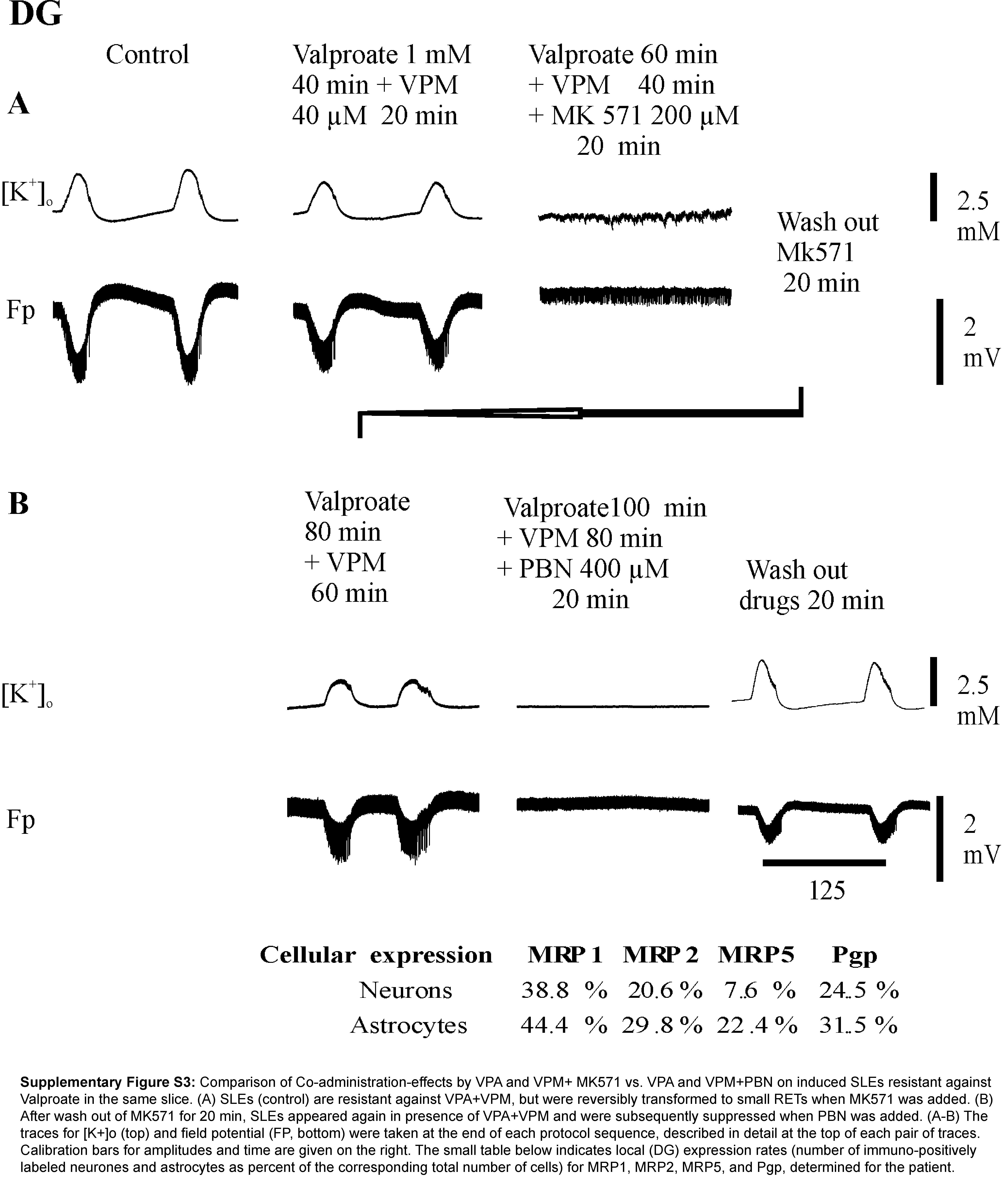

Supplement: Supplementary file 4 [file image_3.tif]

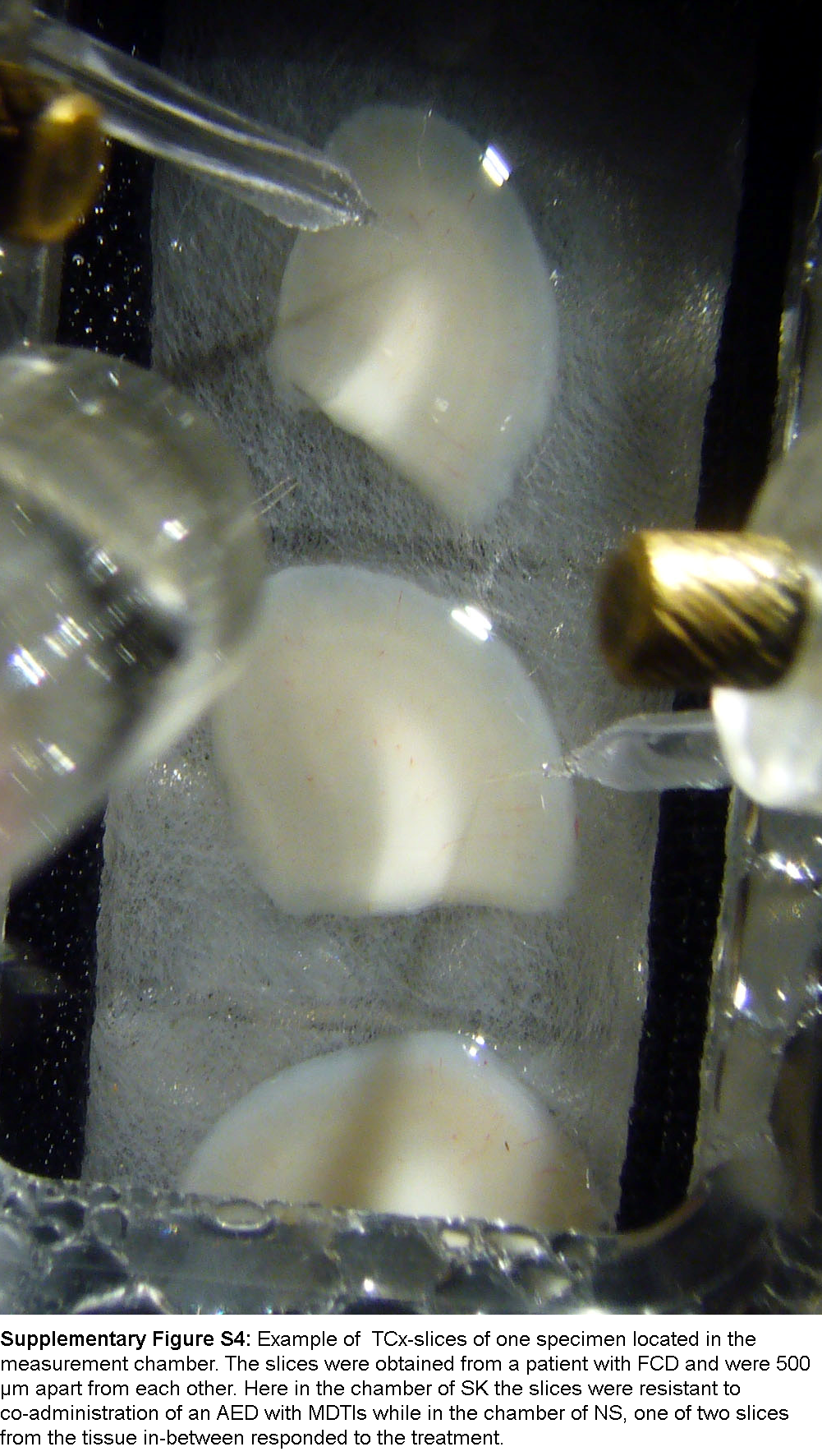

Supplement: Supplementary file 5 [file image_4.tif]
